# Supplementary material for: Entropy-based discrimination between translated Chinese and original Chinese using data mining techniques
Source: PLoS One. 2022 Mar 24;17(3):e0265633. doi: 10.1371/journal.pone.0265633 (PMC8947138; doi:10.1371/journal.pone.0265633)
Supplement: Attachment — Submitted filename: review PONE-D-21-26016.docx [file pone.0265633.s001.docx]

Review PONE-D-21-26016

General Comments: In the present study, Liu et al. tried to discriminate between translated- and non-translated Chinese by using the data-driven machines learning methods, in which they extracted seven entropy-based indicators including character, wordform unigram, wordform bigram, wordform trigram, POS (Part-of-speech) unigram, POS bigram, and POS trigram entropy from two balanced Chinese comparable corpora. In fact, they were separating the translated Chinese from non-translated Chinese by adopting the Support Vector Machines (SVMs), Linear discriminant analysis (LDA), Random Forest (RF), and Multilayer Perceptron (MLP) algorithms. The results showed that SVMs is the most robust and effective classifier. Therefore, I think the method is reliable and the results are novel enough. In addition, the Discuss section is substantial and explains the results well. I think the authors are asking an important question that has not yet been thoroughly addressed in the literature, and I would like to further raise some minor points that deserve clarification at this stage.

1. In the Introduction section, although the literary beginning is good, I consider it may not be appropriate in the academic journal (lines 2-6).

2. The authors’ statement that “Xiao and Yue’s study [28] found that translated Chinese fiction contains a significantly greater mean sentence length than native Chinese fiction, which contradicts previous assumption proposed by Malmkjær [29]”, lines 111-113. The assumption need be showed here.

4. The Discussion and Conclusion section should be separated.

In sum, I think the present paper may make a significant contribution to the literature in the field of translation studies. I suggest a minor revision at this stage.
