# Supplementary material for: Entropy-based discrimination between translated Chinese and original Chinese using data mining techniques
Source: PLoS One. 2022 Mar 24;17(3):e0265633. doi: 10.1371/journal.pone.0265633 (PMC8947138; doi:10.1371/journal.pone.0265633)
Supplement: Attachment — Submitted filename: Response to Reviewers.docx [file pone.0265633.s002.docx]

**Responses to reviews**

We thank the editor and the reviewers for their insightful comments. We are glad that they have found the current research to be valuable, the method used reliable to provide novel results and the discussion substantial to explain the results. In sum, we thank the reviewers for noting that we have filled the research gap by asking an important question that has not yet been thoroughly addressed in the existing literature.

We have further revised the manuscript in light of the review comments. Below, we summarise the major changes we have made to the manuscript, and then we detail our responses to the review comments point by point.

**Revisions**

1. We have rewritten the introduction by deleting the literary beginning which the reviewer consider it may not be appropriate in the academic journal.

2. We have separated discussion and conclusion (originally one section), which have now become two different sections.

**Responses to Reviewer**

*1. In the Introduction section, although the literary beginning is good, I consider it may not be appropriate in the academic journal (lines 2-6).*

Thank you for the reviewer’s comment. We have now deleted the literary beginning and changed it with a different one (see p. 1). The paragraph is given below.

*2. The authors’ statement that “Xiao and Yue’s study [28] found that translated Chinese fiction contains a significantly greater mean sentence length than native Chinese fiction, which contradicts previous assumption proposed by Malmkjær [29]”, lines 111-113. The assumption need be showed here.*

We appreciate the reviewer’s careful reading. We have now revised this sentence as follows (also see lines pp. 3-4).

“Xiao and Yue's study [28] found that translated Chinese fiction contains a significantly greater mean sentence length than native Chinese fiction, which contradicts the previous assumption [29] that the general tendency in translation is to adjust the source text punctuations in order to conform to the target language norms, thus resulting in similar sentence lengths between the source texts and comparable target texts.”

*3. The Discussion and Conclusion section should be separated.*

We have followed the reviewer’s suggestion by separating Discussion and Conclusion. Also note that we have added an extra paragraph to sum up the study. (see pp. 13-15)
